# Effectiveness of emicizumab in preventing bleeding events in severe and moderate hemophilia A: A single‐center experience in Bangladesh

**DOI:** 10.1002/jha2.832

**Published:** 2024-01-09

**Authors:** Sanzina Sadia Tory, Sujan Ghosh, Humayra Nazneen, Nurul Farhad, Salwa Islam, Mohammad Jahid Hasan, Akhil Ranjan Biswas

**Affiliations:** ^1^ Department of Hematology Dhaka Medical College Hospital Dhaka Bangladesh; ^2^ Pi Research Development Center Dhaka Bangladesh; ^3^ Department of Health System Research Tropical Disease and Health Research Center Dhaka Bangladesh

**Keywords:** annualized bleeding rate, efficacy, emicizumab, hemophilia A, prophylaxis, safety

## Abstract

Emicizumab is a monoclonal antibody that bridges activated factor IX (FIX) and factor X (FX) to replace the function of missing activated factor VIII (FVIII) in hemophilia A patients irrespective of FVIII inhibitor status. This study assessed the effectiveness of emicizumab in preventing bleeding episodes in patients with hemophilia A. This observational study included patients with moderate to severe hemophilia A who were undergoing episodic FVIII replacement therapy. The primary endpoint was the difference in annualized bleeding rates (ABR) and the secondary endpoint was the difference in Hemophilia Joint Health Score (HJHS) before and after emicizumab prophylaxis. A total of 30 male hemophilia patients were included, the mean age was 16.7 (SD: ±8.1) years, and most of them had moderate hemophilia A [63.3%]. Before prophylaxis, the median ABR was 48 (interquartile range [IQR]: 35–60), and 93.3% of patients had ABR greater than eight, whereas after prophylaxis the median ABR decreased significantly (median [IQR]: 0 [0.0–0.4], *p* < 0.001), and 56.7% had zero bleeds. ABR was not significantly different in patient with and without FVIII inhibitors. The HJHS scores significantly improved after prophylaxis (10 vs. 2.5, *p* < 0.001). The bleeding events were reduced significantly (23 vs. 0.0, *p* < 0.001), and zero new target joints were reported after prophylaxis. Most of the patients [93.3%] did not face any serious adverse events after prophylaxis. Emicizumab prophylaxis was associated with a significantly lower rate of bleeding events among participants with hemophilia A, regardless of inhibitor status.

## 1 INTRODUCTION

Hemophilia is one of the inherited bleeding disorders with a distinct genetic, epidemiological, biochemical, and clinical profile [1]. Hemophilia is caused by a mutation in the factor VIII (FVIII) gene that causes a deficiency or dysfunction of coagulation clotting factor. A severe clinical bleeding phenotype, evident as spontaneous bleeding, becomes apparent when the FVIII level drops below 1% of the normal range in most hemophilia A patients [2]. Among the prominent indicators of hemophilia A is intra‐articular bleeding, especially in the knee, elbow, and ankle joints. The recurrence of bleeding within joints is the chief contributor to morbidity, gradual and irreversible joint impairment, and the occurrence of hemophilic arthropathy in patients with hemophilia A [3].

Replacement of the missing FVIII with FVIII infusion is used to treat bleeding, which can occur in the muscles, skin, and mucous membranes, as well as joints. Replacement therapy in hemophilia A is related with favorable outcomes, including enhancement of quality of life [4], reduced musculoskeletal complications [4, 5], and lower morbidity and mortality [4, 6]. However, administering episodic FVIII replacements does not change the inherent course of spontaneous bleeding, eventually leading to musculoskeletal damage and other complications due to bleeding. Therefore, the widely accepted practice of using prophylaxis, in which a clotting factor is administered to prevent spontaneous bleeding and sustain protective FVIII plasma trough level, is being adopted for hemophilia patients [7]. The primary concerns in hemophilia A replacement therapy are the development of anti‐FVIII neutralizing antibodies (inhibitors) and the increased treatment workload due to intravenous administration and prevention efforts [8, 9]. Inhibitors arise as the most challenging complication of replacement therapy, affecting as many as 30% of hemophilia A patients and undermining the effectiveness of replacement therapy [10, 11]. Thus, non‐factor replacement treatments for hemophilia A have been developed due to the high treatment burden and lower therapeutic efficacy of FVIII concentrate in hemophilia patients with inhibitors.

The monoclonal antibody and FVIII mimetic antibody, emicizumab, is one of the few registered non‐replacement therapies used worldwide to treat hemophilia A. Emicizumab is a recombinant, humanized, monoclonal bispecific modified immunoglobulin G4 (IgG4) antibody that bridges activated factor IX and factor X to restore the function of missing activated FVIII in hemophilia A [12]. Due to its half‐life lasting around 30 days, emicizumab necessitates less frequent administration via the subcutaneous route. Moreover, routine prophylactic dosing with this agent eliminates the need for regular laboratory monitoring, rendering it a compelling option for prophylactic treatment in individuals with hemophilia A. Because emicizumab lacks any structural homology with FVIII, it is not anticipated to trigger the development of FVIII inhibitors and can function irrespective of the presence of FVIII inhibitors [13]. Emicizumab has demonstrated efficacy in bleeding prevention, reducing annualized bleeding rates (ABRs) [14, 15, 16, 17] and long term administration of emicizumab are well–tolerated with no thromboembolic episodes and no neutralizing anti‐emicizumab antibody appearing during the course of treatment in patients with hemophilia A [17, 18]. Because of improved pharmacokinetic profile and subcutaneous administration, emicizumab is likely to have a direct impact in reducing treatment and disease burden in hemophilia A patients.

**Novelty statement:**

**What is the new aspect of your work?**

Currently, safety and effectiveness of emicizumab have not been evaluated comprehensively in the context of Bangladesh. Therefore, this study was conducted to assess emicizumab's effectiveness in preventing bleeding events in people with hemophilia A.

**What is the central finding of your work?**

Prophylactic treatment with emicizumab has led to significant improvements in this study population. The annualized bleeding rate (ABR) and frequency of bleeding event significantly reduced, irrespective of the FVIII inhibitors status. Moreover, the administration of emicizumab led to an improvement in the Hemophilia Joint Health Score (HJHS) among individuals with hemophilia A.

**What is (or could be) the specific clinical relevance of your work?**

Emicizumab prophylaxis significantly reduced bleeding rates in hemophilia A patients, regardless of FVIII inhibitor status. Emicizumab offers a promising, convenient prophylactic treatment option, potentially improving patient care and adherence while reducing morbidity in hemophilia A patients.

Although studies have been conducted to determine the safety and efficacy of emicizumab in hemophilia A patients, but this was not assessed in our country perspective. Therefore, we conducted this study in Bangladesh to evaluate the effectiveness of emicizumab in treating hemophilia A. The findings of this study will contribute to the literature on the safety and efficacy of emicizumab in treating hemophilia A, and may have implications for future research and clinical practice and provide valuable insights for clinicians and researchers particularly in light of the growing burden of bleeding disorders and the increasing demand for evidence‐based interventions.

## 2 MATERIALS AND METHODS

### 2.1 Study design and participants

This single‐center, observational study was conducted in the hemophilia treatment center (HTC) of a tertiary care hospital for a period of one year. The study protocol was approved by ethical review committee of Dhaka Medical College (ERC‐DMC/ECC/2021/114). A total of 60 hemophilia A patients were approached initially and among them 30 patients who fulfilled the selection criteria were selected. The selection criteria involved patients with severe hemophilia A and moderate hemophilia A with severe bleeding phenotype with ABR of > 8 irrespective of FVIII inhibitor status. Hemophilia individuals who were receiving episodic FVIII replacement therapy, were eligible to participate. To determine the differences in the ABR and hemophilia joint health score (HJHS) in children and adults, the patients aged ≤18 years were considered as children and adolescents and more than 18 years were considered as adults [19]. Participants who had suitable hematological, hepatic, and renal functions were included. Suitable hematologic function was defined as having a platelet count ≥100,000/μL and a hemoglobin level of ≥8 g/dL (4.97 mmol/L). Suitable hepatic function was defined as having a total bilirubin level ≤1.5 times the upper limit of normal (ULN) and both AST (aspartate aminotransferase) and ALT (alanine aminotransferase) levels of less than or equal to three times the ULN at the time of screening. Suitable renal function was defined as having a serum creatinine level ≤2.5 times the ULN and a creatinine clearance of ≥30 mL/min, defined by Cockcroft–Gault formula. Patients showing any clinical signs or have known laboratory or radiographic evidence consistent with cirrhosis were excluded. Top‐of‐form patients were ineligible for the study if they had ongoing/planned immune tolerance induction (ITI) therapy.

All participants and their legal guardians were informed details about emicizumab, study characteristics, and purpose of the study in an easily understandable way. All information regarding the benefits and hazards of the study was delivered to all the participants and their legal guardians, and only those who agreed to participate after written informed consent were included in the study. The research was conducted following the guidelines outlined in the Declaration of Helsinki and adhering to Good Clinical Practice. Information was gathered via an interview utilizing a structured questionnaire.

### 2.2 Study procedure

Before administration of emicizumab prophylaxis therapy, all patients were screened for FVIII inhibitors. Among the 30 patients, eight hemophilia A patients had FVIII inhibitors and 22 had no FVIII inhibitors. The ABR was calculated from the previous 6 months of bleeding history using the formula of the number of reported bleeding events, divided by the number of months in the reporting time window, and multiplied by 12. The joint health was evaluated by HJHS, which incorporates nine parameters: swelling (0–3), duration of swelling (0–1), muscle atrophy (0–2), crepitus on motion (0–2), flexion loss (0–3), extension loss (0–3), joint pain (0–2), strength (0–4) for elbows, knees, and ankles, and a global gait score (0–4). The HJHS scores range from 0 to 20 per joint and the global gait score ranges from 0 to 4, resulting in a total HJHS score from 0 to 124 points. A higher score indicated a worse joint health [20]. Emicizumab prophylaxis was administered by subcutaneous injection. Prophylactic treatment plan consisted of four initial loading doses of 3 mg/kg bodyweight per week, followed by a maintenance dose of 6 mg/kg every 4 weeks for at least 6 months in total at the study site. Individuals who had previously undergone episodic FVIII replacement therapy before joining the study were allowed to continue their usual prophylactic routine until after the second emicizumab loading dose, to prevent bleeding incidents prior to achieving sufficient emicizumab levels. Throughout the study period, patients and their caregivers were properly instructed to keep records of every breakthrough bleeding event and inform the HTC. Patients were followed up regularly and at each visit, detailed history of breakthrough bleeding was recorded regarding site, and severity of bleed, whether spontaneous or traumatic, and what management was taken for bleeding control. Significant bleeding events were managed with FVIII replacement therapy at HTC. For patients with inhibitor, breakthrough bleeding was managed with fresh frozen plasma (FFP) and tranexamic acid, a synthetic derivative of lysine used as an antifibrinolytic to treat major bleeding. Any side effect of the drug was properly evaluated. At each visit, bodyweight was taken for dose adjustment.

### 2.3 Study endpoints

The primary endpoint of this study was to evaluate the percentage reduction of ABR from the baseline to over a period of 6 months. In this context, a 100% reduction of ABR was considered as the elimination or cessation of bleeding events. Secondary endpoints included HJHS and additional bleeding‐related endpoints: number of bleeding events, ABRs with inhibitor status, and presence of target joint bleeding before and after emicizumab prophylaxis. Target joints were defined as major joints (e.g., hip, elbow, wrist, shoulder, knee, and ankle) in which three or more bleeding events occurred over a 6‐month period. We also assessed the differences in the primary and secondary endpoints in children, adolescents, and adults.

### 2.4 Statistical analysis

We used descriptive statistics to demonstrate the information of the participants who were enrolled. Continuous data were expressed as means and standard deviation (SD) or median and interquartile range (IQR) depending on the distribution of data. Categorical variables were expressed as frequencies and percentages. Comparisons of ABRs, number of bleeding events, and HJHS before and after prophylaxis were assessed by Mann–Whitney *U* test and Wilcoxon signed rank test, where *p*‐value less than 0.05 was considered significant.

## 3 RESULTS

### 3.1 Demographic and bleeding characteristics

All 30 male hemophilia A patients received a subcutaneous loading dose of emicizumab at 3 mg/kg once weekly for the initial 4 weeks, and then 6 mg/kg once every 4 weeks as maintenance regimen. Among the hemophilia A patients, the mean age of the patients was 16.7 (SD: ±8.1) years, which ranged from 3.5 to 37 years and the median age of first bleeding event was 7.5 (IQR: 6.0–13.5) months. Most of the patient had moderate hemophilia A [63.3%]. At baseline, the presence of FVIII inhibitor and target joint was 26.7% and 86.7%, respectively, and all the patients took episodic FVIII replacement therapy. The median bleeding event was 23 (IQR: 16–32), and life‐threatening bleeding like intra‐cranial bleeding was reported in 63.6% (Table 1).

**TABLE 1:** Baseline demographics and disease characteristics of hemophilia A patients (*n* = 30).

Variable	*n*	%
**Age (years)**		
Up to 12	7	23.3
13–18	11	36.7
>18	12	40.0
Mean ± SD	16.7 ± 8.14	
**Travel time from residence to HTC, DMCH (h)**		
<2	16	53.3
2–6	9	30
>6	5	16.7
**Age at first bleeding (months)**		
<6	14	46.7
7–12	9	30.0
>12	7	23.3
Median [IQR]	7.5	[6.0–13.5]
**Hemophilia A severity at baseline**		
Moderate	19	63.3
Severe	11	36.7
**Presence of target joint** ^a^		
Yes	26	86.7
No	4	13.3
**Presence of FVIII inhibitors at study entry**		
Yes	8	26.7
No	22	73.3
**Prior treatment**		
Episodic factor replacement	30	100
Regular prophylaxis	0	0
**Bleeding event in the 6 months before study entry**		
Median [IQR]	23 [16–32]	
**Life‐threatening bleeding event (*n* = 22)**		
Intracranial	14	63.6
Intra‐abdominal	6	27.3
Pseudotumor	2	9.1

Abbreviations: FVIII, coagulation factor VIII; HTC, hemophilia treatment center; IQR, interquartile range.

^a^ Target joints were defined as major joints (e.g., hip, elbow, wrist, shoulder, knee, and ankle) in which at least three bleeding events occurred over a period of 24 weeks.

### 3.2 Primary endpoint

**FIGURE 1:**
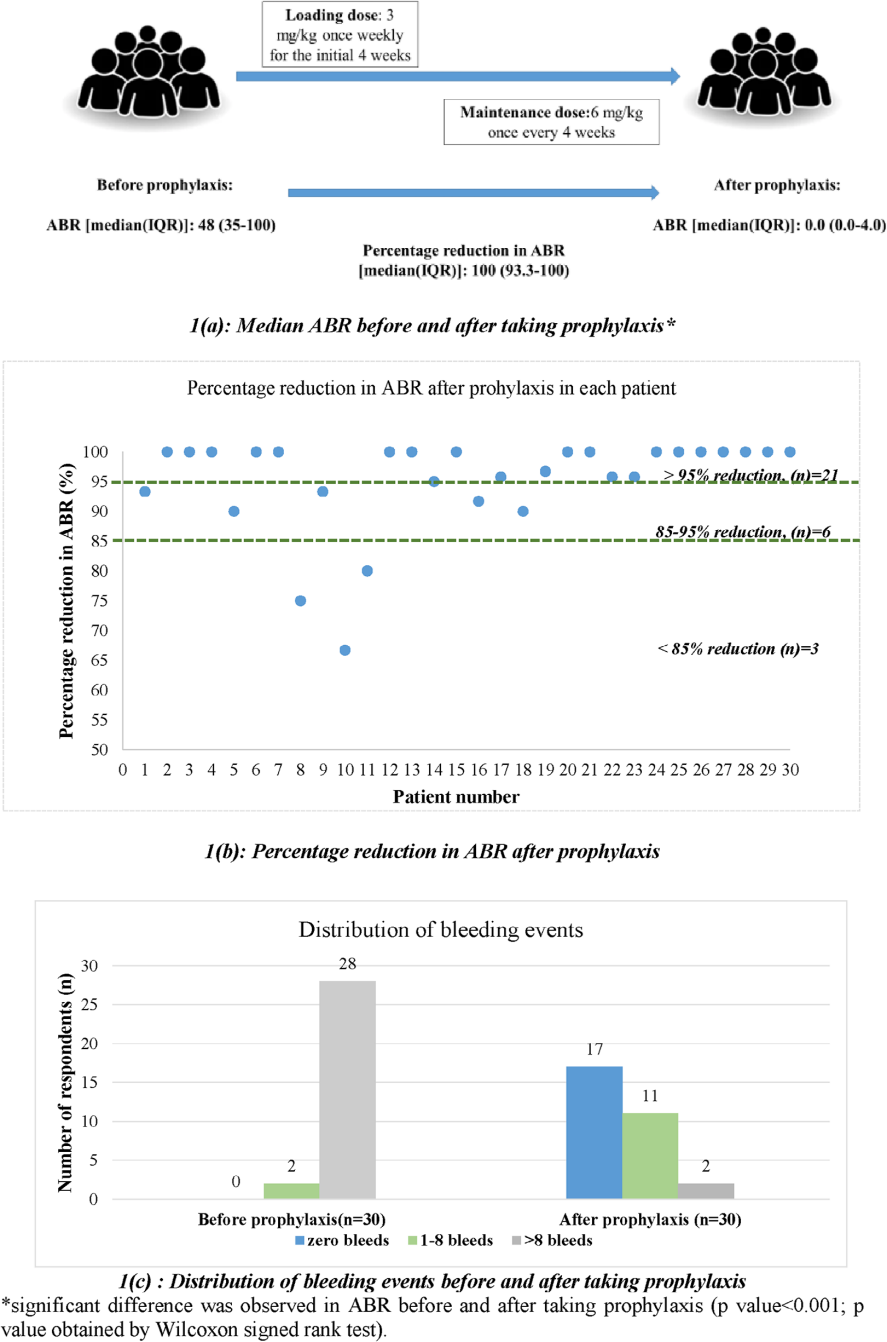
Comparison of endpoints before and after emicizumab prophylaxis (*n* = 30).

Afteremicizumab, 70% (*n* = 21) had achieved greater than 95% reduction in ABR, of which 17 patients had achieved 100% reduction in ABR. Before emicizumab prophylaxis, the median ABR was 48 [35–60], and 28 [93.3%] patients had more than eight ABR, whereas after prophylaxis, the ABR reduced significantly (median [IQR]: 0.0 [0.0–0.4], *p* < 0.001), and 17 [56.7%] patients had zero bleeds while 11 [36.7%] patients had one to eight bleeds, and two [6.6%] patients had more than eight bleeds (Figure 1).

### 3.3 Secondary endpoints

When assessing the HJHS before administration of emicizumab, the median HJHS score was 10.0 [7.7–17.2], and half of the patients [50.0%] had an HJHS from 1 to 10, while nine [30.0%] patients had HJHS from 11 to 20. After prophylaxis, the score was reduced to 2.5 [0.0–8.2], and 11 [36.7%] patients had an HJHS of 0 while, 13 [43.3%] patients had a HJHS from 1 to 10. Wilcoxon signed rank test showed that the HJHS of the patients significantly reduced after prophylaxis (*p* < 0.001) (Table 2).

**TABLE 2:** Comparison of HJHS, ABR with inhibitor status, and bleeding events before and after emicizumab prophylaxis (*n* = 30).

	Before prophylaxis *n* (%)	After prophylaxis *n* (%)	*p*‐Value^*^
**HJHS**			
Median [IQR]	10.0 [7.7–17.2]	2.5 [0.0–8.2]	<0.001
0	1 (3.3)	11 (36.7)	
1–10	15 (50)	13 (43.3)	
11–20	9 (30)	4 (13.3)	
21–30	3 (10)	2 (6.7)	
>30	2 (6.7)	0 (0)	
ABR with inhibitor, median [IQR]	48.0 [33.0–60.0]	2.0 [0.0–11.5]	<0.001
ABR without inhibitor, median [IQR]	48.0 [33.0–60.0]	1.0 [0.0–4.0]	<0.001
Bleeding events, median [IQR]	23 [16–32]	0.0 [0.0–2.0]	<0.001

Abbreviations: ABR, annualized bleeding rate; HJHS, Hemophilia Joint Health Score; IQR, interquartile range.

* *p*‐Value obtained by Wilcoxon signed rank test.

After emicizumab prophylaxis, the bleeding events were significantly reduced (*p* < 0.001). Among these 13 patients who had new bleeding event during emicizumab regimen, 43.3% had only joint bleeding, 15.4% had both gum bleeding and joint bleeding, 7.7% had both hematuria and joint bleeding, and 7.7% patients had muscle bleeding (Table S1) and zero new target joint bleeding was observed. The ABR decreased significantly in both patients with and without inhibitors (*p* < 0.001). There was no significant difference in ABR between patients with and without inhibitor after emicizumab prophylaxis (Table S2).

### 3.4 Efficacy of emicizumab prophylaxis on children and adult patients of hemophilia A

After prophylaxis, the median ABR and bleeding event was 0.0 [0.0–2.0] and 0.0 [0.0–2.0], respectively, in children and adolescents. There was no significant difference in the ABR and bleeding event between children and adult patients of hemophilia A after taking emicizumab. The HJHS score did not differ significantly between adults and children after taking emicizumab [4.5 (0.25–17) vs. 1.5 (0.0–5.25), *p* = 0.185] (Table 3).

**TABLE 3:** Comparison of ABR, HJHS, and bleeding events in children/adolescents and adults after emicizumab prophylaxis (*n* = 30).

	Children and adolescents *n* = 18 Median [IQR]	Adults *n* = 12 Median [IQR]	*p*‐Value^*^
ABR	0.0 [0.0–2.0]	0.0 [0.0–5.5]	0.172
Percentage reduction in ABR	100 [95.8–100]	94.6 [90.4–100]	0.134
HJHS	1.5 [0.0–5.25]	4.5 [0.25–17]	0.185
Bleeding events	0.0 [0.0–2.0]	0.0 [0.0–4.5]	0.917

Abbreviations: ABR, annualized bleeding rate; HJHS, Hemophilia Joint Health Score; IQR, interquartile range.

* *p*‐Value obtained by Mann–Whitney *U* test.

### 3.5 Adverse events

Table 4 demonstrates that 27 [90%] patients did not face any adverse events after emicizumab prophylaxis, whereas one [3.3%] patient had an allergic reaction and another patient had sleep disturbance and palpitation. Allergic reaction was managed by antihistamines, and sleep disturbance and palpitation were managed by counseling (Table 4).

**TABLE 4:** Summary of adverse events in patients taking emicizumab (*n* = 30).

ABR reduction	*n*	%
No adverse events	27	90
Allergic reaction	1	3.3
Sleep disturbance	1	3.3
Palpitation	1	3.3

Abbreviation: ABR, annualized bleeding rate.

## 4 DISCUSSION

The results from the present study suggested that emicizumab could offer satisfactory protection against bleeds and clinically significant bleeding control irrespective of FVIII inhibitor status, consistent with previous study findings [14, 15, 21]. The ABR and HJHS of the patients were significantly reduced after prophylaxis in comparison to prior no prophylaxis. After prophylaxis, majority [50%] of patients had a 100.0% reduction of ABR. There was no significant difference between patients with or without inhibitor regarding ABR. Most of the patients did not face any adverse events. Additionally, despite majority of the participants presented with target joints at baseline, 100% of patients reported zero treated target joint bleeds after receiving emicizumab prophylaxis, which was higher than the published studies of both standard and extended half‐life FVIII prophylaxis regimens [3, 22, 23], indicating that emicizumab prophylaxis given subcutaneously can provide effective bleed prevention.

This is the first study to investigate the efficacy and safety of emicizumab prophylaxis in adults and adolescents with hemophilia A, both with and without FVIII inhibitors in Bangladesh, a lower middle‐income country where only 5.5% of hemophilia A patients receive prophylactic treatment and most of the patients cannot afford standard treatment and are receiving inadequate on‐demand therapy for hemophilia A [24]. Majority of the participants included in the study were above the age of 18 years, had spontaneous bleeding episodes of more than 20, were mostly moderate cases of hemophilia A, and did not have FVIII inhibitors or a record of ITI therapy or prior use of bypassing agents, which was anticipated and consistent with demographic information on the hemophilia A population in previous studies [15, 25].

A total of 50% of the participants who received emicizumab prophylaxis had zero bleeding events during the study along with an ABR of 0.0 (IQR: 0–0.4). These positive outcomes confirm previously reported results of a phase‐1 clinical trial [15], and are consistent with a series of HAVEN studies carried out among children, adults, and adolescents with hemophilia A. In the HAVEN 1 study, weekly emicizumab was well tolerated and linked to low rate of ABR in adults and adolescents (ABR: 2.9) with hemophilia A and FVIII inhibitors, and a significant number of participants experienced zero treated bleeds [63%]. Similarly, in HAVEN 2, pediatric patients with hemophilia A and FVIII inhibitors who received weekly emicizumab achieved an even lower ABR [0.3], with a large proportion reporting zero treated bleeds [77%] [15, 21]. The reduction in the ABR and the higher percentage of participants experiencing zero bleeds are likely linked to a lower occurrence of joint damage and a decreased frequency of target joints in the pediatric population when compared to the adult and adolescent populations [26]. However, in our study the reduction in ABR and HJHS score was not significantly different in children, adolescents, and adults after taking emicizumab, which may be attributable to the fact that the sample size in this study was small to demonstrate the differences. Additionally, in our study after 6 months of emicizumab prophylaxis, the percentage reduction in ABR was 66.7%–100%, which was similar to reductions reported in other studies [27, 28]. The substantial decrease in bleeding incidents among patients using emicizumab prophylaxis, as opposed to those without any prophylactic treatment, can be attributed to a notable enhancement in the quality of life and overall health status of patients diagnosed with hemophilia A [29]. We found no evidence that the efficacy of emicizumab was affected by FVIII inhibitor status (*p* = 0.166), although this conclusion should be made cautiously because of the small number of patients with inhibitors in this study.

Emicizumab prophylaxis led to clinically relevant improvement in joint health. The median HJHS decreased from 10.0 [7.7–17.2] to 2.5 [0.0–8.2). No patient had deterioration in joint health score and zero target joint was observed after 6 months of emicizumab, indicating persistent joint health benefits with emicizumab prophylaxis. Similarly, the HAVEN 3 clinical trial reported a significant improvement in joint health scores in adolescents and young adults and in those with target joints after 48 weeks of emicizumab [12]. Safety investigations showed that most of the patients [93.3%] did not face any adverse events after prophylaxis, whereas one [3.3%] patient had a mild allergic reaction at injection site and another patient had sleep disturbance and palpitation. All the events were mild and had spontaneous resolution. No serious adverse events and thromboembolic events or thrombotic microangiopathies were reported, which was similar to findings to previous studies [16, 18].

The study's scope was constrained by the limited number of patients, potentially impacting the outcomes of the endpoints. Additionally, information regarding treated bleeds was based on self‐reporting of treated bleeds by patients; therefore, the evaluation of the presence of a bleed was subjective. Nevertheless, by incorporating both bleeds and treated spontaneous bleeds as endpoints, the study offered insights into the bleeding patterns of these patients. Further research is necessary to define the utilization of emicizumab in children under the age of 1, including those with previously untreated hemophilia A.

## 5 CONCLUSION

The study results suggested that emicizumab prophylaxis administered subcutaneously following four loading doses weekly, led to a significant reduction in the bleeding rate compared with no prophylaxis among hemophilia A patients, regardless of the presence of FVIII inhibitors. Following prophylaxis, more than half of the participants had zero bleeds, and joint health improved significantly. Emicizumab presents a potential weekly subcutaneous prophylactic treatment for individuals with hemophilia A. This treatment option has the potential to improve patient care, promote the acceptance of, and adherence to, effective prophylaxis, serving as an impactful approach to reduce morbidity in individuals with hemophilia A.

## AUTHOR CONTRIBUTIONS

Conceptualization: Sanzina Sadia Tory, Nurul Farhad, Akhil Ranjan Biswas, and Humayra Nazneen. Formal analysis: Mohammad Jahid Hasan, Salwa Islam, and Sanzina Sadia Tory. Investigation: Sanzina Sadia Tory, Nurul Farhad, Akhil Ranjan Biswas, Humayra Nazneen, Sujan Ghosh, Mohammad Jahid Hasan, and Salwa Islam. Methodology: Nurul Farhad, Akhil Ranjan Biswas, Humayra Nazneen, Sujan Ghosh, Mohammad Jahid Hasan, and Salwa Islam. Resources: Nurul Farhad, Humayra Nazneen, Sujan Ghosh, Mohammad Jahid Hasan, and Salwa Islam. Supervision: Sanzina Sadia Tory and Akhil Ranjan Biswas. Writing: Salwa Islam, Akhil Ranjan Biswas, and Mohammad Jahid Hasan. All authors read and approved the final version of the manuscript.

## CONFLICT OF INTEREST STATEMENT

The authors declare they have no conflicts of interest.

## FUNDING INFORMATION

The authors have no support or funding to report.

## ETHICS STATEMENT

The study was approved by the Ethical Review Committee of Dhaka Medical College. Informed written consent was obtained from all eligible participants who agreed to participate. The authors declare no human subjects were harmed and the procedures followed were in accordance with the ethical standards and regulations established by the Helsinki Declaration of the World Medical Association.

## PATIENT CONSENT STATEMENT

The authors have confirmed patient consent statement is not needed for this submission.

## CLINICAL TRIAL REGISTRATION

The authors have confirmed clinical trial registration is not needed for this submission.

## Supporting information

Supporting Information

## Data Availability

Patient‐level data will be available on request from the corresponding author.

## References

[1] Srivastava A , Brewer AK , Mauser‐Bunschoten EP , Key NS , Kitchen S , Llinas A , et al. Guidelines for the management of hemophilia. Haemophilia. 2013;19(1):1–47. 10.1111/j.1365-2516.2012.02909.x 22776238

[2] Mahlangu J . Emicizumab for the prevention of bleeds in hemophilia A. Expert Opin Biol Ther. 2019;19(8):753–761. 31150297 10.1080/14712598.2019.1626370

[3] Valentino LA , Mamonov V , Hellmann A , Quon DV , Chybicka A , Schroth P , et al. A randomized comparison of two prophylaxis regimens and a paired comparison of on‐demand and prophylaxis treatments in hemophilia A management. J Thromb Haemost. 2012;10(3):359–367. 22212248 10.1111/j.1538-7836.2011.04611.x PMC3488301

[4] Wyrwich KW , Krishnan S , Auguste P , Poon J‐L , von Maltzahn R , Yu R , et al. Changes in health‐related quality of life with treatment of longer‐acting clotting factors: results in the A‐LONG and B‐LONG clinical studies. Haemophilia. 2016;22(6):866–872. 27385432 10.1111/hae.12987

[5] Carcao M , Hilliard P , Escobar MA , Solimeno L , Mahlangu J , Santagostino E . Optimising musculoskeletal care for patients with haemophilia. Eur J Haematol. 2015;95:11–21. 26679393 10.1111/ejh.12581

[6] Witmer CM . Low mortality from intracranial haemorrhage in paediatric patients with haemophilia. Haemophilia. 2015;21(5):359–363. 10.1111/hae.12716 26010533

[7] Berntorp E , Andersson NG . Prophylaxis for hemophilia in the era of extended half‐life factor VIII/factor IX products. Semin Thromb Hemost. 2016;42(5):518–525. 27096762 10.1055/s-0036-1571315

[8] Von Mackensen S , Kalnins W , Krucker J , Weiss J , Miesbach W , Albisetti M , et al. Haemophilia patients’ unmet needs and their expectations of the new extended half‐life factor concentrates. Haemophilia. 2017;23(4):566–574. 28370896 10.1111/hae.13221

[9] Mahlangu J , Cerquiera M , Srivastava A . Emerging therapies for haemophilia—global perspective. Haemophilia. 2018;24:15–21. 10.1111/hae.13510 29878661

[10] Bardi E , Astermark J . Genetic risk factors for inhibitors in haemophilia A. Eur J Haematol. 2015;94(s77):7–10. 25560788 10.1111/ejh.12495

[11] DeKoven M , Karkare S , Lee WC , Kelley LA , Cooper DL , Pham H , et al. Impact of haemophilia with inhibitors on caregiver burden in the United States. Haemophilia. 2014;20(6):822–830. 25273645 10.1111/hae.12501

[12] Kiialainen A , Niggli M , Kempton CL , Castaman G , Chang T , Paz‐Priel I , et al. Effect of emicizumab prophylaxis on bone and joint health markers in people with haemophilia A without factor VIII inhibitors in the HAVEN 3 study. Haemophilia. 2022;28(6):1033–1043. 35905294 10.1111/hae.14642 PMC9796488

[13] Scott LJ , Kim ES . Emicizumab‐kxwh: first global approval. Drugs. 2018;78:269–274. 29357074 10.1007/s40265-018-0861-2

[14] Mahlangu J , Oldenburg J , Paz‐Priel I , Negrier C , Niggli M , Mancuso ME , et al. Emicizumab prophylaxis in patients who have hemophilia A without inhibitors. N Engl J Med. 2018;379(9):811–822. 30157389 10.1056/NEJMoa1803550

[15] Oldenburg J , Mahlangu JN , Kim B , Schmitt C , Callaghan MU , Young G , et al. Emicizumab prophylaxis in hemophilia A with inhibitors. N Engl J Med. 2017;377(9):809–818. 28691557 10.1056/NEJMoa1703068

[16] Pipe SW , Shima M , Lehle M , Shapiro A , Chebon S , Fukutake K , et al. Efficacy, safety, and pharmacokinetics of emicizumab prophylaxis given every 4 weeks in people with haemophilia A (HAVEN 4): a multicentre, open‐label, non‐randomised phase 3 study. Lancet Haematol. 2019;6(6):295–305. 10.1016/S2352-3026(19)30054-7 31003963

[17] Shima M . Emicizumab prophylaxis overcomes factor VIII inhibitors in hemophilia A. J Pediatr. 2017;190:287–290. 10.1016/j.jpeds.2017.08.053 29144254

[18] Rodriguez‐Merchan EC , Valentino LA . Emicizumab: review of the literature and critical appraisal. Haemophilia. 2019;25(1):11–20. 30431213 10.1111/hae.13641

[19] UNICEF . Investing in a safe, healthy and productive transition from childhood to adulthood is critical. SAGEEP; 2012. p. 73–87.

[20] Kuijlaars IAR , van der Net J , Feldman BM , Aspdahl M , Bladen M , de Boer W , et al. Evaluating international Haemophilia Joint Health Score (HJHS) results combined with expert opinion: options for a shorter HJHS. Haemophilia. 2020;26(6):1072–1080. 33058441 10.1111/hae.14180 PMC7821332

[21] Young G , Liesner R , Chang T , Sidonio R , Oldenburg J , Jiménez‐Yuste V , et al. A multicenter, open‐label phase 3 study of emicizumab prophylaxis in children with hemophilia A with inhibitors. Blood. 2019;134(24):2127–2138. 31697801 10.1182/blood.2019001869 PMC6908828

[22] Giangrande P , Andreeva T , Chowdary P , Ehrenforth S , Hanabusa H , Leebeek FWG , et al. Clinical evaluation of glycoPEGylated recombinant FVIII: efficacy and safety in severe haemophilia A. Thromb Haemost. 2017;117(2):252–261. 27904904 10.1160/TH16-06-0444

[23] Mahlangu J , Powell JS , Ragni MV , Chowdary P , Josephson NC , Pabinger I , et al. Phase 3 study of recombinant factor VIII Fc fusion protein in severe hemophilia A. Blood. 2014;123(3):317–325. 24227821 10.1182/blood-2013-10-529974 PMC3894491

[24] Hossain MS , Mosabbir AA . Haemophilia in South Asia: a perspective from Bangladesh. Haemophilia. 2022;28(1):18–19. 10.1111/hae.14445 34676626

[25] Islam MN , Biswas AR , Nazneen H , Chowdhury N , Alam M , Banik J , et al. Clinical profile and demographic characteristics of moderate and severe hemophilia patients in a tertiary care hospital of Bangladesh. Orphanet J Rare Dis. 2022;17(1):1. 10.1186/s13023-021-02091-x 35804421 PMC9264493

[26] Hanley J , McKernan A , Creagh MD , Classey S , McLaughlin P , Goddard N , et al. Guidelines for the management of acute joint bleeds and chronic synovitis in haemophilia: a United Kingdom Haemophilia Centre Doctors’ Organisation (UKHCDO) guideline. Haemophilia. 2017;23(4):511–520. 28370924 10.1111/hae.13201

[27] Shima M , Sidonio RF . Substitution therapy. Haemophilia. 2021;27(S3):53–59. 32558019 10.1111/hae.14072

[28] Young G , Callaghan M , Dunn A , Kruse‐Jarres R , Pipe S . Emicizumab for hemophilia A with factor VIII inhibitors. Expert Rev Hematol. 2018;11(11):835–846. 30278802 10.1080/17474086.2018.1531701

[29] Wyrwich KW , Krishnan S , Poon JL , Auguste P , von Maltzahn R , Yu R , et al. Interpreting important health‐related quality of life change using the Haem‐A‐QoL. Haemophilia. 2015;21(5):578–584. 25828456 10.1111/hae.12642

