# Supplementary material for: Effectiveness of emicizumab in preventing bleeding events in severe and moderate hemophilia A: A single‐center experience in Bangladesh
Source: EJHaem. 2024 Jan 9;5(1):39–46. doi: 10.1002/jha2.832 (PMC10887364; doi:10.1002/jha2.832)
Supplement: Supplementary file 1 — Supporting Information [file JHA2-5-39-s001.docx]

**Supplementary table-1**

**Table 1 Distribution of patients by bleeding events after prophylaxis (n=30)**

| **Bleeding events** | **n** | **%** |
| --- | --- | --- |
| **Absent** | 17 | 56.7 |
| **Present** | 13 | 43.3 |
| Only Joint bleeding | 9 | 69.2 |
| Gum bleeding+ Joint bleeding | 2 | 15.4 |
| Hematuria+ Joint bleeding | 1 | 7.7 |
| Muscle bleeding | 1 | 7.7 |

**Supplementary table-2**

**Table 2 Comparison of ABR after prophylaxis among patients with or without inhibitors (n=30)**

| **ABR** | **With inhibitor**  **n=8**  **n(%)** | **Without inhibitor**  **n=22**  **n(%)** | **p value** |
| --- | --- | --- | --- |
| **Percent reduction of ARB** |  |  | 0.166* |
| <95% | 4(15.2) | 4(50) |  |
| 95%-99% | 5 (22.7) | 0 (0.0) |  |
| 100% | 13 (59.1) | 4 (50.0) |  |
| **ABR [median(IQR)]** | 2.0 (0.0-11.5) | 1.0 (0.0-4.0) | 0.534** |

*p value obtained by Fisher’s exact test

**p value obtained by Wilcoxon signed rank test
